# Pneumatosis Intestinalis in COVID-19: Case Series

**DOI:** 10.7759/cureus.10991

**Published:** 2020-10-16

**Authors:** Kelvin Wong, Dae Hyeon Kim, Sameer Khanijo, Aleksandr Melamud, Gulrukh Zaidi

**Affiliations:** 1 Pulmonary and Critical Care Medicine, Northwell Health North Shore University Hospital/Long Island Jewish Medical Center, Manhasset, USA

**Keywords:** covid-19, severe acute respiratory syndrome coronavirus 2, pneumatosis cystoides intestinalis, intestinal perforation, interleukin-6

## Abstract

Objective: To describe the clinical characteristics and outcomes of patients with coronavirus disease 2019 (COVID-19) who developed pneumatosis intestinalis (PI).

Methods: This case series was conducted in intensive care units at two large tertiary care centers within the Northwell Health System, located in New York State. Patients were included if they were identified as having confirmed COVID-19 as well as pneumatosis intestinalis from March 16, 2020 to July 31, 2020. Patient demographics, clinical characteristics, vasopressor use, anticoagulation use, opiate use, paralytic use, COVID-19 treatment regimen, serum lactate, arterial pH, serum bicarbonate, subsequent intervention, and outcomes during hospitalization were collected.

Results: A total of nine patients were identified. Average serum lactate was 4.33 mmol/L at time of diagnosis. Portal venous gas (56%) and bowel dilation (56%) were common radiographic findings. Subsequent morbidity (increased vasopressor requirements - 67%, acute kidney injury - 67%, increased oxygen requirements - 44%) and mortality (78%) were high. PI occurred despite a majority of patients being on anticoagulation (78%). Interleukin-6 (IL-6) inhibitors were commonly administered (56%) prior to development of PI.

Conclusion: Pneumatosis intestinalis in COVID-19 is clinically significant, with high morbidity and mortality, and is also likely underdiagnosed.

## Introduction

As of September 15, 2020, the number of confirmed coronavirus disease 2019 (COVID-19) cases have exceeded 29 million worldwide, resulting in almost one million deaths. As scientific and medical communities continue efforts to elucidate how the responsible pathogen, severe acute respiratory syndrome coronavirus 2 (SARS-CoV-2), can affect the human body, we have discovered many, often unexpected, ways it can cause harm.

Pneumatosis intestinalis (PI) refers to the presence of gas within the intestinal wall. Many aspects of this condition are poorly understood, including etiology, pathogenesis, and clinical implication. Reported etiologies have been widely varied, including gastroenteritis [1], chronic obstructive pulmonary disease, bowel obstruction, idiopathic [2], etc. While it may be a benign, incidental finding, it may also herald a life-threatening condition such as intestinal ischemia. Previous studies have shown that elevated lactate, vasopressor use, small bowel dilation, peritonitis, and abdominal pain are predictive factors for clinically significant PI [3-5].

PI has been previously reported in COVID-19 patients, but it has been limited to three single case reports and a review of abdominal imaging findings in COVID-19 patients. In their review of 134 patients, Bhayana et al. reported that abdominal computed tomography (CT) detected pneumatosis or portal venous gas in four of 20 critically ill patients (20%) [6]. In some of these patients, surgical findings showed unusual yellow discoloration of bowel and bowel infarction while pathology demonstrated ischemic enteritis, patchy necrosis, and fibrin arteriolar thrombi.

Here, we report a case series of PI in patients with COVID-19 infection and describe their clinical characteristics and outcomes.

## Materials and methods

Data was collected retrospectively from the electronic medical record (EMR), Enterprise Sunrise Clinical Manager-Allscripts, for patients with confirmed COVID-19 and pneumatosis intestinalis admitted to North Shore University Hospital and Long Island Jewish Medical Center, two large tertiary care centers within the Northwell Health System, from March 16, 2020 to July 15, 2020. This case series was approved by the Northwell Institutional Review Board (IRB), as minimal-risk research using data collected for routine clinical practice and waived the requirement for informed consent (IRB number 20-0200).

A confirmed case of COVID-19 was defined as a positive laboratory test for SARS-CoV-2 from real-time reverse transcription polymerase chain reaction (RT-PCR) assay of a respiratory specimen. Pneumatosis intestinalis was identified by abdominal CT. We collected data on patient demographics, comorbidities, time from symptom onset to PI diagnosis, CT imaging findings, concomitant lab values (serum lactate, arterial pH, serum bicarbonate), subsequent intervention, COVID-19 treatment regimen, vasopressor use, anticoagulation use, opiate use, paralytic use, and patient outcomes (change in vasopressor requirements, change in oxygen requirements, development of acute kidney injury, resolution of PI and in-hospital mortality).

## Results

A total of nine patients were included in our case series. The patients were predominantly male (seven patients, 77.8%) with an average age of 54.3 years. Three patients were Hispanic (33.3%) with the rest equally distributed among White, Black, and Asian ethnicities (two patients, 22.2%). The most common comorbidity was obesity (six patients, 66.7%), followed by hypertension (three patients, 33.3%), diabetes (two patients, 22.2%), and prior history of cerebrovascular accident or transient ischemic attack (two patients, 22.2%). The average time of symptom onset to PI diagnosis was 22.2 days and the average time of admission to PI diagnosis was 13.7 days (Table 1).

**Table 1:** Patient Demographics and Characteristics Note: Values are expressed as number (percentage) unless otherwise indicated

Demographics	Total Cohort (n = 9)
Age (years)	
Mean [Range]	54.3 [36 – 65]
Gender	
Male	7 (78)
Female	2 (22)
BMI (kg/m2)	
Mean [Range]	32.8 [23.1 - 56.4]
Normal (18.5 to < 25)	2 (22)
Overweight (25 to < 30)	2(22)
Obese (>30)	5 (56)
Race	
Hispanic	3 (33)
White	2 (22)
Asian	2 (22)
Black	2 (22)
Comorbidities	
HTN	3 (33)
DM	2 (22)
Prior CVA	2 (22)
CKD	1 (11)
Asthma	1 (11)
Medications at time of diagnosis	
Anti-coagulation	7 (78)
Opiate	7 (78)
Vasopressor	5 (56)
Paralytic	3 (33)
COVID-19 Specific Medications	
Steroids	6 (67)
Anti-Il 6	5 (55)
Plasma	3 (33)
Plaquenil	3 (33)
Anti-Il 1	2 (22)
Azithromycin	2 (22)

Indications for abdominal CT were variable. The majority of scans were performed as part of an infectious workup (four patients, 44.4%). Other indications included workup for elevated serum lactate (two patients, 22.2%), abdominal distension (two patients, 22.2%), and confirmation of incidental findings on chest CT (one patient, 11.1%).

Most patients had pneumatosis of both small and large bowel (six patients, 66.7%), while two patients (22.2%) had only small bowel involvement and one patient (11.1%) had only large bowel involvement. Portal venous gas was present in five patients (55.5%). Bowel dilation was also present in five patients (55.5%), of which three patients (33.3%) had dilated loops of both large and small bowel, while two patients (22.2%) had dilated loops of small bowel alone (Table 2).

**Table 2:** Clinical Data

Clinical Data	Total Cohort (n = 9)
Days from symptom onset to diagnosis of pneumatosis	
Mean [Range]	22.2 [4 – 47]
Days from admission to diagnosis of pneumatosis	
Mean [Range]	13.7 [0 – 31]
Laboratory Findings	
Lactate (mmol/L)	
Mean (SD)	4.33 (3.64)
Arterial pH	
Mean (SD)	7.22 (0.13)
Bicarbonate (mmol/L)	
Mean (SD)	19.33 (5.83)
Radiology Findings (n,%)	
Portal Venous Gas	5 (56)
Dilated Bowel Loops	5 (56)
Small Bowel Only	2 (22)
Large Bowel Only	0 (0)
Both	3 (33)
Interventions (n,%)	
NPO	9 (100)
Antibiotics	7 (78)
Stopped Bowel Regimens	3 (33)
Total Parenteral Nutrition	2 (22)
Surgical intervention	1 (11)
Outcome (n,%)	
Increased Vasopressor Requirements	6 (67)
Acute Kidney Injury	6 (67)
Inpatient Deaths	7 (78)
Discharged	2 (22)
Days from pneumatosis diagnosis to death	
Mean [Range]	6.43 [0 – 16]

At the time of CT diagnosis of PI, the average serum lactate was 4.33 mmol/L (range 0.8 to 12.4). Average arterial pH was collected for eight patients, with an average value of 7.22 (range 7.06 to 7.39). Average serum bicarbonate was 19.33 mmol/L (range 9 to 30) (Table 2).

The majority of patients were on full anticoagulation (seven patients, 77.8%) with four patients on argatroban and three patients on unfractionated heparin. Most were also on continuous opiate infusions for sedation (seven patients, 77.8%). Of the patients in our series, five (55.6%) were on vasopressor medications. A minority of patients were on paralytics (three patients, 33.3%) (Table 1).

COVID-19-specific therapy included a variety of different regimens. The most commonly used medications were steroids (six patients, 66.7%) followed by IL-6 inhibitors (five patients, 55.6%), convalescent plasma (three patients, 33.3%), hydroxychloroquine (three patients, 33.3%), interleukin-1 inhibitors (two patients, 22.2%) and azithromycin (two patients, 22.2%). Two patients (22.2%) received no COVID-19-specific therapy (Table 1).

After diagnosis of PI, one patient (11.1%) underwent an exploratory laparotomy, which was done emergently at bedside and revealed necrotic bowel. All other patients were managed conservatively with bowel rest (100%), empiric antibiotics to cover intestinal flora (77.8%), and discontinuation of bowel regimens (33.3%). Two patients (22.2%) were also started on total parenteral nutrition (TPN) (Table 2).

Following diagnosis of PI, six patients (66.7%) developed increased vasopressor requirements, six patients (66.7%) developed acute kidney injury, and four patients (44.4%) developed increased oxygen requirements. Resolution was demonstrated on interval imaging in two patients (22.2%). In terms of mortality, seven patients expired (77.8%) and two were discharged (22.2%). For those who expired, the average time from PI diagnosis to death was 6.4 days (Table 2).

## Discussion

To our knowledge, this study is the largest reported case series of pneumatosis intestinalis in COVID-19 patients. In our series, 77.8% of patients who were diagnosed with PI died. A majority of these patients also developed significant morbidity after the diagnosis of PI, such as increased vasopressor requirements (66.7%) and acute kidney injury (66.7%). For those who died, average time to death after PI diagnosis was 6.4 days while their average length of stay was 20.4 days (Table 2). These findings suggest that the development of PI is a poor prognostic factor in COVID-19.

Based on pre-COVID-19 studies on predictive factors for pathologic PI, our results support that the diagnosis of PI in COVID-19 patients is of clinical significance. Multiple studies have shown that serum lactate is the most consistent predictor of clinically significant PI. One study showed that lactate levels greater that 2.0 mmol/L at time of diagnosis has been associated with over 80% mortality [4]. In our case series, this was true in 77.8% of patients (odds ratio = 21.7). The presence of small bowel dilation has also been shown to be a predictor, which was present in 55.6% of our patients (odds ratio = 1.3).

The pathophysiology of this process is unclear as many aspects of COVID-19 remain unknown. Recent COVID-19 autopsy studies have shown a 40% prevalence of deep venous thrombosis, presence of microthrombi in multiple organ systems, as well evidence of intestinal endothelitis suggesting both macro and microvascular clotting predisposition [7-9]. However, the majority of patients in our case series developed PI despite therapeutic anticoagulation (77.8%), suggesting that there may be other contributing factors. The usage of opiate medications (77.8%) and paralytics (33.3%) with subsequent decreased gut motility can in theory increase intraluminal pressures and cause intestinal distention, increasing risk for wall injury.

While each patient had indications for emergent surgical exploration based on pre-COVID-19 studies, risks of surgical intervention were deemed to outweigh benefits for most. The only patient with surgical intervention had undergone exploratory laparotomy the same day of PI diagnosis and died the next day. Two patients (22.2%) had resolution of PI without surgical intervention, one of which survived to discharge. It is unclear if early surgical intervention would be beneficial.

This manifestation of disease has significant implications on possible treatment modalities of interest. Recently, “cytokine storm” has been purported to be partly responsible for the high morbidity and mortality seen in COVID-19. Interleukin-6 inhibitors are being investigated for their potential in stemming hyperinflammation and its deleterious effects. Tocilizumab, an Il-6 inhibitor, has a known elevated risk of intestinal perforation [10,11]. Intestinal perforation has also been reported with sarilumab, another inhibitor. In our group, five patients (55.6%) had received an IL-6 inhibitor with an average time from administration to PI diagnosis of 11.4 days. Of these five patients, four received tocilizumab and one received sarilumab. The effect of receiving these medications on their development of PI is unclear.

## Conclusions

We describe a case series of COVID-19 patients who developed pneumatosis intestinalis. We believe it is a clinically significant manifestation of disease that is likely underdiagnosed. Clinical suspicion should be high in COVID-19 patients with clinical decompensation, rising serum lactate, or worsening abdominal exam. Further surgical and pathology data is necessary to determine exact pathophysiology.
